# Supplementary material for: A cross-sectional survey of false beliefs about COVID-19 and their association with vaccine hesitancy and uptake in the United States and China
Source: PLOS Glob Public Health. 2026 Feb 25;6(2):e0005939. doi: 10.1371/journal.pgph.0005939 (PMC12935220; doi:10.1371/journal.pgph.0005939)
Supplement: Attachment — Submitted filename: PLOS GPH Author response to reviewer feedback blk RD.docx [file pgph.0005939.s002.docx]

**We thank the reviewers and editors for their constructive feedback. We have made every effort to incorporate your suggestions. Additionally, we added departmental affiliations for each author to the manuscript. Given the statical technicalities of this paper, we felt it might be helpful to identify our third author as a statistician.**

PGPH-D-25-01987
A cross-sectional survey of false beliefs about COVID-19 and their association with vaccine hesitancy and uptake in the United States and China
PLOS Global Public Health

**Response to Editor and Reviewers**

Journal Requirements:

1. Please amend your detailed Financial Disclosure statement. This is published with the article. It must therefore be completed in full sentences and contain the exact wording you wish to be published.

1. Please clarify all sources of funding (financial or material support) for your study. List the grants (with grant number) or organizations (with url) that supported your study, including funding received from your institution. 
2. State the initials, alongside each funding source, of each author to receive each grant.
3. State what role the funders took in the study. If the funders had no role in your study, please state: “The funders had no role in study design, data collection and analysis, decision to publish, or preparation of the manuscript.”
4. If any authors received a salary from any of your funders, please state which authors and which funders.

If you did not receive any funding for this study, please simply state: “The authors received no specific funding for this work.”

**Author Response: Financial Disclosure statement added to the manuscript before the Abstract.**

2. Please send a completed 'Competing Interests' statement, including any COIs declared by your co-authors. If you have no competing interests to declare, please state "The authors have declared that no competing interests exist". Otherwise please declare all competing interests beginning with the statement "I have read the journal's policy and the authors of this manuscript have the following competing interests:"

**Author Response: Competing Interests’ statement added to the manuscript after the Financial Disclosure statement.**

3. Please provide separate figure files in .tif or .eps format.

For more information about figure files please see our guidelines:  
<https://journals.plos.org/globalpublichealth/s/figures> 
<https://journals.plos.org/globalpublichealth/s/figures#loc-file-requirements>

**Author Response: We are submitting for separate .tif files labeled Figure1a, Figure1b, Figure2a, Figure2b.**

4, If the reviewer comments include a recommendation to cite specific previously published works, please review and evaluate these publications to determine whether they are relevant and should be cited. There is no requirement to cite these works unless the editor has indicated otherwise. 

Reviewers' comments:

Reviewer's Responses to Questions

**Comments to the Author**

1. Does this manuscript meet PLOS Global Public Health’s [publication criteria](https://www.google.com/url?q=https://track.editorialmanager.com/CL0/https:%252F%252Fjournals.plos.org%252Fglobalpublichealth%252Fs%252Fcriteria-for-publication/1/010f019931b7c8c4-6f212103-cef6-4375-9bcb-16ff21f5ebce-000000/71i4iHcS8nPqefNA5S22xgklLA-TF6g0YEdi6TNowZs%3D228&source=gmail-imap&ust=1758080865000000&usg=AOvVaw3gcofEW4nO16XvpvP8h9EO)? Is the manuscript technically sound, and do the data support the conclusions? The manuscript must describe methodologically and ethically rigorous research with conclusions that are appropriately drawn based on the data presented.

Reviewer #1: Partly

Reviewer #2: Yes

2. Has the statistical analysis been performed appropriately and rigorously?

Reviewer #1: No

Reviewer #2: Yes

3. Have the authors made all data underlying the findings in their manuscript fully available (please refer to the Data Availability Statement at the start of the manuscript PDF file)?

The [PLOS Data policy](https://www.google.com/url?q=https://track.editorialmanager.com/CL0/https:%252F%252Fjournals.plos.org%252Fglobalpublichealth%252Fs%252Fdata-availability/1/010f019931b7c8c4-6f212103-cef6-4375-9bcb-16ff21f5ebce-000000/eipeCuG9sxaRcT3LvBIabwKO-30pOUQdHysfp9OY5Dk%3D228&source=gmail-imap&ust=1758080865000000&usg=AOvVaw0ZV01JlY9a89BR9KDeMINS) requires authors to make all data underlying the findings described in their manuscript fully available without restriction, with rare exception. The data should be provided as part of the manuscript or its supporting information, or deposited to a public repository. For example, in addition to summary statistics, the data points behind means, medians and variance measures should be available. If there are restrictions on publicly sharing data—e.g. participant privacy or use of data from a third party—those must be specified.

Reviewer #1: Yes

Reviewer #2: Yes

4. Is the manuscript presented in an intelligible fashion and written in standard English?

PLOS Global Public Health does not copyedit accepted manuscripts, so the language in submitted articles must be clear, correct, and unambiguous. Any typographical or grammatical errors should be corrected at revision, so please note any specific errors here.

Reviewer #1: Yes

Reviewer #2: Yes

5. Review Comments to the Author

Please use the space provided to explain your answers to the questions above. You may also include additional comments for the author, including concerns about dual publication, research ethics, or publication ethics. (Please upload your review as an attachment if it exceeds 20,000 characters)

Reviewer #1: General comment
The study provides an important insight on COVID-19 vaccine acceptance and uptake in two countries with diverse context. This study is of interest. But there are some issues related to data analysis and results presentation.

Introduction
L48-51 the objective of the study objective typically appears at the end of the introduction.

**Author Response: We removed the purpose of the study from this section and now only appears in the Present Research section.**

L60 I think it should be ‘higher proportion of vaccinated’.

**Author Response: This statement indicates that there are a greater number of overall people in China who are vaccinated and a higher percent of the overall population who are vaccinates.**

L113-116 I think this section is similar to this section L123-125 I suggest to harmonize.

**Author Response: We agree that this is repetitive. We removed the hypothesis from the first paragraph in The Present Research and discuss it only in second paragraph.**

Methods
Kindly specify how you tested the normality of continuous variables such as age in the data analysis subsection.

**Author Response: The standard modern statistical guidance, as supported by current literature, is that formal normality testing (e.g., Shapiro-Wilk or Kolmogorov-Smirnov tests) is not a necessary prerequisite for choosing statistical methods for variables like age in the context of common group comparisons (e.g., t-tests, ANOVA) or correlations.**

**This is primarily for two reasons. Parametric tests like the t-test are robust to violations of normality, particularly with sample sizes above ~30-40 per group (due to the Central Limit Theorem). Our sample size of around 900 (around at least 450 from each country) is more than sufficient for this robustness. Further, normality tests are overly sensitive in large samples, often detecting trivial deviations from normality that have no practical impact on the results of parametric tests. Conversely, they are underpowered in small samples, failing to detect important violations. There are several references that support this and we have listed one below:**

**Ghasemi, A., & Zahediasl, S. (2012). Normality tests for statistical analysis: a guide for non-statisticians. International Journal of Endocrinology and Metabolism, *10*(2), 486–489.**

**Moreover, we would like to clarify that in multiple linear regression, the assumption of normality applies to the model residuals/errors rather than to the independent variables. Normality of errors ensures the validity of p-values and confidence intervals. Predictor variables (independent variables) can follow any distribution (e.g., highly skewed age, bimodal distributions). Errors in a linear regression model are computed as follows:**

$$\boldsymbol{e}_{\boldsymbol{i}}\mathbf{=}\boldsymbol{y}_{\boldsymbol{i}}\mathbf{-}{\hat{\boldsymbol{y}}}_{\boldsymbol{i}}\mathbf{,}\boldsymbol{i}\mathbf{=}\boldsymbol{1}\mathbf{,...,}\boldsymbol{n}$$

**where** $\boldsymbol{n}$ **is the number of subjects in the analysis,** $\boldsymbol{e}_{\boldsymbol{i}}$ **is the estimated error in prediction for** $\boldsymbol{i}^{\boldsymbol{th}}$ **subject,** $\boldsymbol{y}_{\boldsymbol{i}}$ **is the *actual* value of the response variable predicted in the model and** ${\hat{\boldsymbol{y}}}_{\boldsymbol{i}}$ **is the *predicted* value of the response variable for** $\boldsymbol{i}^{\boldsymbol{th}}$ **subject. The predicted value of** $\boldsymbol{y}_{\boldsymbol{i}}$ **is obtained as follows:**

$${\hat{\boldsymbol{y}}}_{\boldsymbol{i}}\mathbf{=}\boldsymbol{b}_{\boldsymbol{0}}\mathbf{+}\boldsymbol{b}_{\boldsymbol{1}}\boldsymbol{x}_{\boldsymbol{1}\boldsymbol{i}}\mathbf{+....+}\boldsymbol{b}_{\boldsymbol{p}}\boldsymbol{x}_{\boldsymbol{pi}}$$

**where** $\boldsymbol{x}_{\boldsymbol{1}\boldsymbol{i}}$ **is the value of the predictor** $\boldsymbol{X}_{\boldsymbol{1}}$ **measured on subject** $\boldsymbol{i}$**,** $\boldsymbol{x}_{\boldsymbol{2}\boldsymbol{i}}$ **is the value of the predictor** $\boldsymbol{X}_{\boldsymbol{2}}$ **measured on subject** $\boldsymbol{i}$**, and so on. Here** $\boldsymbol{p}$ **is the number of predictors in the model.** $\boldsymbol{b}_{\boldsymbol{0}}$ **is the sample estimate of the true (population) y-intercept** $\boldsymbol{\beta}_{\boldsymbol{0}}$**,** $\boldsymbol{b}_{\boldsymbol{1}}$ **is the sample estimate of the true (population) regression coefficient for predictor variable** $\boldsymbol{X}_{\boldsymbol{1}}$**,** $\boldsymbol{b}_{\boldsymbol{2}}$ **is the sample estimate of the true (population) regression coefficient for predictor variable** $\boldsymbol{X}_{\boldsymbol{2}}$ **and so on. Thus, as we can see, residuals are a measure of how much discrepancy exists between actual and predicted values of the response variable for the subjects in the study. Hence, it is natural that the assumptions of normality, independence and homoscedasticity (constant variation) in a linear regression model are applicable and checked for the errors and not the predictors. There are several standard statisticial texts that confirm this, such as Gelman and Hill (2006) on page 45, Draper and Smith (1998) on page 141, etc.**

**Therefore, while performing regressions, we did not test age (or other continuous predictors) for normality. Instead, as recommended in standard regression practice, we assessed the model residuals to verify the normality assumption using the Shapiro-Wilk’s normality test and Breusch-Pagan test for diagnosing heteroscedasticity in the errors. When those assumptions were shown to be violated, we performed a log transformation on the dependent variable and calculated robust standard errors (which were then used to calculate robust t test statistics and p-values) to mitigate these issues, which is another common practice in linear regression modelling. The justification for this course of action can also be found in Gelman and Hill (2006) and Wooldridge(2012).**

Results
Kindly provide the response rate of this study.

**Author Response: This information is included in the first paragraph of the Results section.**

The title 1 is not adequately written, kindly remove the redundant “and” in this title.

**Author Response: Removed**

For P value I suggest to write the whole value for example instead of .08, write 0.08.

**Author Response: Revised**

Kindly specify in footnote the meaning of t and X or X2 used in table 1. Is there a difference between X and X2 in this table?

**Author Response: X was an error and was corrected to X^2^**

Not all reader might adequately identify that the number in parenthesis is the degree of freedom. I suggest the author to explain that in the footnote for more clarity. In summary specify in the footnote what is being presented in column US/China comparisons.

**Author Response: We described the analysis in a footnote for the table. In addition, we removed degrees of freedom from the report in the table as it is clear from the number of countries.**

What do you mean by M and SD in table 1.

**Author Response: Defined in table 1.**

You could consider specifying after the name of the variable this ‘n (%)’ and adjust the content and position of number and percentage accordingly and then remove the repeating % and N=.
Usually for sample, the number of participants is noted n not N.

**Author Response: Table modified to reflect this suggestion.**

The interpretation of table 1 is sufficient. Kindly provide in one or two sentence a brief description of the results presented in table 1.

**Author Response: Summary sentence added after Table 1 is mentioned in the text.**

The total number and percentage are matching across variables nor for the total number of participants. If there were no response kindly specify that in the table as for table 2.

**Author Response: Row added to Table 1 for nonbinary/non-response participants.**

In table 2 the significance of results are not specified in the interpretation. Kindly adjust this.

**Author Response: Significance added.**

Were all participants eligible for a booster dose as it is typically required for those who have already completed the initial number of required doses for the vaccine received.
Somebody which is not vaccinated should not be typically include as potential booster eligible individuals.

**Author Response:**

**We have clarified how we defined booster COVID-19 doses in this study. The reviewer correctly points out that the true definition of a booster dose is additional doses in a prescribed schedule, that typically dose not include annual vaccination. For the purposes of this analysis, any COVID-19 vaccine dose administered after 2021 was considered a booster, since that was the language used when those shots became available and we felt that would be more familiar to participants.**

I think there might a bias in the interpretation of the booster hesitancy. How did you handle in this study the potential bias resulting from the partial vaccination against COVID-19 which might also result from hesitancy to received the second doses due concern about vaccine adverse effects or other reasons?

**Author Response: See previous comment about clarification of booster hesitancy, which should address this question as well. Furthermore, we chose to examine the hesitancy to the original shot and subsequent shots separately, for exactly the reason the reviewer pointed out. There were additional concerns about the side-effects at that time and less concern about the adverse effects from COVID infections.**

In the interpretation of table 3 kindly specify in the significance of results as your comparing two countries.

**Author Response: Significance added.**

There is no need to repeat the ¨% after the value as this is specified in the heading of each column.
In table 4.

**Author Response:**

**We would prefer to leave them in as we feel it makes the table easier to read. However, we will defer to the copy editor’s decision.**

It is known the best model is the lowest AIC’. why presenting multiple models with AIC instead of selecting and presenting the best model at the multivariate analysis.

**Author Response: In both tables we included only the best fit models, and deleted the other models.**

Why not presenting the univariate regression analysis, near the multivariate analysis to enable the reader to identify the influence of the modelling or adjustment of potential covariates?

**Author Response: We added Table 4, which presents the associations between each predictor variables and each outcome variable.**

In table 4 I still have concerns about the validity of booster status assessment.
**Author Response: See previous comment about clarification of booster hesitancy.**

The age being a continuous variable its OR is not typically interpretable instead we should consider categorizing the age in other to interpret the OR. For continuous variable the sign and the value of the coefficient is used to interpret is influence in the probability of the dependent variable.

**Author Response:** **The Odds Ratio (OR) for a continuous variable is directly and meaningfully interpretable (Agresti, 2013; Hosmer, Lemeshow and Sturdivant, 2013; Nahhas, 2024). For a continuous variable x, a basic interpretation of the magnitude of** $\boldsymbol{\beta}$ **is that the odds multiply by** $\boldsymbol{e}^{\boldsymbol{\beta}}$ **for every one unit increase in x. Therefore, our reported OR for age can be interpreted as: *’For each additional year of age, the odds of being vaccinated change by [OR-1]*100%.’ Moreover, the standard practice in modern epidemiology and statistics is to keep continuous variables continuous to preserve statistical power and avoid the pitfalls of arbitrary categorization discussed below in detail. The prevailing consensus in modern statistical methodology is that categorizing a continuous variable like age is not only unnecessary but is actively discouraged because it wastes information, reduces statistical power, and can introduce bias (Altman & Royston, 2006; Harrell,2015; Naggara et. al, 2011). Therefore, we believe that presenting the OR for age as a continuous variable is not only interpretable but is also the more methodologically rigorous approach.**

Kindly check the Y axis of figure 1. (probability). How did you obtain the probability of receiving the vaccine for each participant in other the generate the figure 1.

**Author Response: There are numerous examples of finding predicted probabilities from a logistic regression model in statistical literature (James, Witten, Hastie, & Tibshirani, 2021; Hosmer Jr, Lemeshow, & Sturdivant, 2013). For reference, the estimated regression model is written as:**

$$\boldsymbol{log}\left( \frac{\hat{\boldsymbol{p}}}{\boldsymbol{1}\mathbf{-}\hat{\boldsymbol{p}}} \right)\mathbf{=}\boldsymbol{2.0996}\mathbf{+}\boldsymbol{0.2307}\boldsymbol{scale}\left( \boldsymbol{age} \right)\mathbf{+}\boldsymbol{0.4612}\boldsymbol{Gender}\mathbf{-}\boldsymbol{0.6064}\boldsymbol{SES}\mathbf{+}\boldsymbol{0.3836}\boldsymbol{scale}\left( \boldsymbol{vacacc} \right)\mathbf{+}\boldsymbol{5.6559}\boldsymbol{country}\mathbf{-}\boldsymbol{0.1997}\boldsymbol{scale}\left( \boldsymbol{misinfo} \right)\mathbf{-}\boldsymbol{0.7906}\boldsymbol{country}\mathbf{*}\boldsymbol{scale}\left( \boldsymbol{misinfo} \right)\mathbf{,}$$

**where** $\hat{\boldsymbol{p}}$ **is the estimated probability of getting vaccinated for a particular subject. Taking exponential on both sides of the equation above and solving for** $\hat{\boldsymbol{p}}$**, we get:**

$$\hat{\boldsymbol{p}}\mathbf{=}\frac{\boldsymbol{exp}\left( \boldsymbol{A} \right)}{\boldsymbol{1}\mathbf{+}\boldsymbol{exp}\left( \boldsymbol{A} \right)}$$

**where** $\boldsymbol{A}\mathbf{=}\boldsymbol{2.0996}\mathbf{+}\boldsymbol{0.2307}\boldsymbol{scale}\left( \boldsymbol{age} \right)\mathbf{+}\boldsymbol{0.4612}\boldsymbol{Gender}\mathbf{-}\boldsymbol{0.6064}\boldsymbol{SES}\mathbf{+}\boldsymbol{0.3836}\boldsymbol{scale}\left( \boldsymbol{vacacc} \right)\mathbf{+}\boldsymbol{5.6559}\boldsymbol{country}\mathbf{-}\boldsymbol{0.1997}\boldsymbol{scale}\left( \boldsymbol{misinfo} \right)\mathbf{-}\boldsymbol{0.7906}\boldsymbol{country}\mathbf{*}\boldsymbol{scale}\left( \boldsymbol{misinfo} \right)$ **(say).**

**In this way, the probability of obtaining a vaccine can be easily calculated for a particular subject by plugging in the values of the predictor variables for particular subject.**

**The interaction plot in Figure 1 was generated using the plot_model() function from the sjPlot package (Lüdecke, 2018), which internally uses the ggeffects package (Lüdecke, 2018) to compute marginal effects.**

**The values on the y-axis represent the predicted probabilities of the outcome (vaccination status). These probabilities were derived by the R package in a two-step process:**

(1) **Linear Prediction: For each value of the predictors specified in the terms argument (misinfo and countryR), the function calculated the linear predictor (log-odds) using the coefficient estimates from the Firth-penalized logistic regression model (logistf). All other covariates in the model (age, gender, SES, vaccine access) were held constant at their mean (for continuous variables) or reference level (for categorical variables).**
(2) **Inverse-Logit Transformation: The resulting log-odds values were then transformed into probabilities using the standard inverse logit function:** $\boldsymbol{p}\mathbf{=}\boldsymbol{exp}\left( \boldsymbol{A} \right)\mathbf{/}\left( \boldsymbol{1}\mathbf{+}\boldsymbol{exp}\left( \boldsymbol{A} \right) \right)$**, where** $\boldsymbol{A}$ **represents the linear predictor defined earlier.**

What is the aim of measuring the interaction of some independent variables in these models?

**Author Response: Testing for an interaction between country and misinformation was an important component of our analysis, and we decided to test for their significance for reasons related to both statistical and theoretical. The statistical justification for including interaction terms in each model is to avoid model misspecification by omitting significant interaction terms (Gelman & Hill, 2007; Hosmer et. al, 2013; Wooldridge, 2016). When a true interaction exists in the population, forcing the effect of a predictor (e.g., Misinformation) to be the same across all levels of another predictor (e.g., Country) results in a biased estimate of the main effects. Beyond the statistical necessity, testing for significance of interaction effects is aimed at directly addressing one of our central research questions of whether the national context (US vs. China) changes the impact of misinformation on vaccine-related outcomes. The finding that the interaction is significant for initial vaccine hesitancy but not for the other models is, in itself, a key result.**

The use of eb might not be appropriate in linear regression. The exponentiate is typically used in logistic regression modelling. The author could present the coefficient with its sign that is used to interpret the weight and the direction of influence of the independent variable as it increases or decreases in the model.

**Author Response: The use of exponentiated coefficients is in fact appropriate and widely accepted in linear regression models when the dependent variable is modeled on the log scale (Gelman, Hill, & Vehtari, 2021; Wooldridge, 2012). In our model, the response variable is specified as log(y) where** $\boldsymbol{y}$ **is vaccine hesitancy measure or booster hesitancy measure. In this setting, the interpretation of coefficients is well established: if** $\boldsymbol{log}\left( \boldsymbol{y} \right)\mathbf{=}\boldsymbol{\beta}_{\boldsymbol{0}}\mathbf{+}\boldsymbol{\beta}_{\boldsymbol{1}}\boldsymbol{X}_{\boldsymbol{1}}\mathbf{+...+}\boldsymbol{\beta}_{\boldsymbol{p}}\boldsymbol{X}_{\boldsymbol{p}}\mathbf{+}\boldsymbol{\epsilon}$**, where** $\boldsymbol{\epsilon}$ **is the error term, then a one-unit increase in** $\boldsymbol{X}_{\boldsymbol{1}}$ **is associated with an expected multiplicative change of** $\boldsymbol{exp}\left( \boldsymbol{\beta}_{\boldsymbol{1}} \right)$ **in** $\boldsymbol{y}$**, holding other variables contant. This is distinct from logistic regression, where the log-odds are modeled and exponentiated coefficients yield odds ratios. Presenting only the sign of the coefficient** $\boldsymbol{\beta}$**’s for** $\boldsymbol{log}\left( \boldsymbol{y} \right)$ **would describe the direction of influence on the logarithm of the outcome, which is not intuitively meaningful. Exponentiating the coefficient** $\boldsymbol{exp}\left( \boldsymbol{\beta} \right)$**’s allows for a direct and practical interpretation on the original scale of the data, which is a significant advantage of using a log transformation. There is a note at the bottom of the revised Table 5 describing the interpretation of this estimate.**

Other models present with low R2 (21% or 19%) instead the model with 85% best explain the dependent variable and the interpretation should emphasize in that model to avoid confusing the readers with multiple less reliable information.

**Author Response: In both tables we included only the best fit models, and deleted the other models.**

Why is the sample size varying from table 4 to table 5. Kindly clarify this.

**Author Response: We added a statement to the planned analysis section clarifying that each analysis only included the participants who responded to the outcome variable.**

Specify the in Figure 2 in the Y and X axis vaccine hesitancy score or booster hesitancy score or covid-19 false beliefs score.

**Author Response: The Y axes are labeled as Vaccine Hesitancy and Booster Hesitancy, respectively, and the X axes are labeled as COVID-19 False Beliefs.**

Discussion
Consider discussing the fact that despite the covid-19 false belief score is high in some participants in both countries, the US participants were consistently more hesitant or less likely to receive the vaccine.

**Author Response: These findings are discussed in the second and third paragraphs of the discussion.**

Conclusion
The conclusion does nor report the main findings from the study. The comparison between countries is not reported. The mains independent variables explaining vaccine uptake or acceptance are not mentioned. Kindly suggest some strategy to combat false belief in these countries especially in US.

**Author Response:**

**The main findings are reported in the beginning of the Discussion section (lines 370 to 385) and specific strategies to combat are included in the Implications sub-section of the Discussion (lines 399-403). As the Conclusion is also a sub-section of the Discussion section, we would prefer to leave those statements in their original location to reduce repetition.**

References
Kindly comply the referencing style ”PLOS ONE” of the journal.

**Author Response: Referencing style changed to Vancouver with brackets for in-text citations.**

Reviewer #2:

1. There is a need for a definition of terms. What is vaccine hesitancy? What is vaccine refusal? What are 'false beliefs'?

**Author Response: In the first paragraph of the Introduction, we clarify that hesitancy refers to discomfort, uncertainty, or negative attitudes toward vaccination, while refusal refers to the behavior of not vaccinating. False beliefs are defined as the belief that misinformation is accurate.**

2. "While there are many factors." Use an alternative phrase here, as you have used this one earlier.

**Author Response: Phrase replaced with “Although many factors may”**

3. "We predicted that vaccine hesitancy and false beliefs about COVID-19 and the vaccine would be higher in the US than in China." This hypothesis seems to have a specified direction. Are you planning to conduct a one-tailed test?

**Author Response: We used a two-tailed test as it is a stricter statistical criterion.**

4. Could the regulation of the internet in China also affect people's response to the survey?

**Author Response: Yes. We address this in the section Media Regulations and Misinformation Spread in the US and China**

5. How was the sample size determined? Inclusion and exclusion criteria? How do you ensure that there is no multiple response from a single individual? How much was the compensation?

**Author Response: We used CloudResearch, which is an online research platform that manages participant recruitment and data quality for academic studies (statement added to Methods). Our sample size was determined by our research funds budget. CloudResearch required participants to be at least 18 years old located in the places selected by the researcher (in our case 50% from the US and 50% from China). All participants have unique IDs in CloudResearch to ensure that there are no duplicate responses. Compensation is based on minimum wage standards of the country and are not specifically disclosed to researchers.**

6. Any linguistic qualification by these authors? This is important to ensure the meanings of the questions are preserved during translation. Fluency may not be enough to ensure accurate translation and backtranslation.

**Author Response: The practice of translation and back-translation by bilingual fluent individuals is common in survey research. We included a citation for this practice Brislin, R. W. (1970). Back-Translation for Cross-Cultural Research. *Journal of Cross-Cultural Psychology*, *1*(3), 185-216.** [**https://doi.org/10.1177/135910457000100301**](https://doi.org/10.1177/135910457000100301)**, which has been cited over 10,000 times. Additionally, or fourth author (who did the back translation) is a social psychologist trained in survey design.**

7. I think it will be helpful to describe how the variables were assessed better. State the 6-point Likert scale and what each point represents. Do this for all the variables. Also, be clear on the dependent and the independent variables in the methods section.

**Author Response: We have revised the methods section to include this content.**

8. It is good practice to state the approval number of the protocol.
**Author Response: Added to Research Ethics and Consent.**

9. "A two-sided p-value =0.05." How will you use a two-sided p-value when your hypothesis was one-sided?
**Author Response: We used a two-sided p-value because it is a stricter criterion.**

10. What is the response rate?

**Author Response: Because data was collected through CloadResearch (see comment #5 for detail on platform), we do not have data on how many potential participants the study was made available to. We do know how participants did not complete 80% if the study or did not pass all three attention checks. These participants were removed from the sample. In Results section: There were 573 respondents from the US and 567 respondents from China. Participants who did not complete 80% of the survey (N_US_ = 93, N_China_ = 103), or did not pass all three attention checks (N_US_ = 26, N_China_ = 8) were removed from the dataset, resulting in 454 US participants and 456 Chinese participants.**

11. What is a geo-political location? Are you referring to the political climate in the US and China? Can you expatiate on this a bit more and relate it directly to your work?

**Author Response: The phrase geo-political location is confusing and we deleted geo-political and simply used location.**

12. Could the method of recruitment of participants be a limitation?

**Author Response: Certainly. We added the following statement to Limitations: Our data was collected through the participant recruitment platform CloudResearch, and our sample is unlikely to be fully representative of US and Chinese populations.**

6. PLOS authors have the option to publish the peer review history of their article ([what does this mean?](https://www.google.com/url?q=https://track.editorialmanager.com/CL0/https:%252F%252Fjournals.plos.org%252Fglobalpublichealth%252Fs%252Feditorial-and-peer-review-process%2523loc-peer-review-history/1/010f019931b7c8c4-6f212103-cef6-4375-9bcb-16ff21f5ebce-000000/yBE2NfyTYjJvAsI4k2yEGGNNsZPggHds7PiLrd-_U7M%3D228&source=gmail-imap&ust=1758080865000000&usg=AOvVaw38cPR8o7xn09qxusd15FvT)). If published, this will include your full peer review and any attached files.


**Do you want your identity to be public for this peer review?** If you choose “no”, your identity will remain anonymous but your review may still be made public.


For information about this choice, including consent withdrawal, please see our [Privacy Policy](https://www.google.com/url?q=https://track.editorialmanager.com/CL0/https:%252F%252Fwww.plos.org%252Fprivacy-policy/1/010f019931b7c8c4-6f212103-cef6-4375-9bcb-16ff21f5ebce-000000/nl3XNUB7lVIN7eIia0gMkJTg5ZGICD15jk-1U07Zfn0%3D228&source=gmail-imap&ust=1758080865000000&usg=AOvVaw2BELnS54HKvnVhUYsadQGz).

Reviewer #1: **Yes:** Fabrice Zobel Lekeumo Cheuyem

Reviewer #2: **Yes:** Charles Olomofe

[NOTE: If reviewer comments were submitted as an attachment file, they will be attached to this email and accessible via the submission site. Please log into your account, locate the manuscript record, and check for the action link "View Attachments". If this link does not appear, there are no attachment files.]

While revising your submission, please upload your figure files to the Preflight Analysis and Conversion Engine (PACE) digital diagnostic tool, <https://pacev2.apexcovantage.com/>. PACE helps ensure that figures meet PLOS requirements. To use PACE, you must first register as a user. Registration is free. Then, login and navigate to the UPLOAD tab, where you will find detailed instructions on how to use the tool. If you encounter any issues or have any questions when using PACE, please email PLOS at [figures@plos.org](mailto:figures@plos.org). Please note that Supporting Information files do not need this step.
