# Supplementary material for: A cross-sectional survey of false beliefs about COVID-19 and their association with vaccine hesitancy and uptake in the United States and China
Source: PLOS Glob Public Health. 2026 Feb 25;6(2):e0005939. doi: 10.1371/journal.pgph.0005939 (PMC12935220; doi:10.1371/journal.pgph.0005939)
Supplement: Attachment — Submitted filename: Response to Reviewers blk.docx [file pgph.0005939.s003.docx]

6. Review Comments to the Author

Please use the space provided to explain your answers to the questions above. You may also include additional comments for the author, including concerns about dual publication, research ethics, or publication ethics. (Please upload your review as an attachment if it exceeds 20,000 characters)

Reviewer #1: General comment
The authors have done a great revision in their manuscript to improve its quality. However, there are some points that were not adequately addressed. Find below my few remaining comments.

**Thank you for taking the time to review our revised manuscript. We have addressed the remaining comments below.**

Results
I am not convinced with the justification of not performing any simple test to assess the normality and only assume based on the sample size. You can specify that as a limitation of this study.

**We apologize for the lack of clarity in our previous revision/response. In multiple linear regression, the assumption of normality applies to the model residuals/errors rather than to the independent variables. Therefore, while performing regressions we assessed the model residuals to verify the normality assumption using the Shapiro-Wilk’s normality test and Breusch-Pagan test for diagnosing heteroscedasticity in the errors. We neglected to add this to the manuscript in the previous revisions and have now done so (lines 211-213). In the previous version we only reported the outcome of these tests and did not indicate the tests themselves.**

I might not have been able to see the response rate in the first paragraph but only number.

**Response rates (percents) were added (line 220-221).**

For P value I suggest to write the whole value for example instead of .08, write 0.08. this was not applied as recommended.

**All exact p-values listed in tables and text now have a leading zero.**

Somebody which is not vaccinated should not be typically include as potential booster eligible individuals.
I think the author should acknowledge the fact that the analysis did not take into consideration the fact that booster hesitancy included also those who have been previously hesitant to the initial shot of vaccine.

**Thank you for reiterating this concern. We updated the language on measuring booster uptake and hesitancy (line 156-162) to more carefully address this concern.**

I agree with this explanation but it should be clearly specified in the methodology. “1) Linear Prediction: For each value of the predictors specified in the terms argument (misinfo and countryR), the function calculated the linear predictor (log-odds) using the coefficient estimates from the Firth-penalized logistic regression model (logistf). All other covariates in the model (age, gender, SES, vaccine access) were held constant at their mean (for continuous variables) or reference level (for categorical variables).”

**Thank you for this clarification. The following statement was added to the planned analysis section to explain the predicted probability calculation for both logistic regression models (lines 204-207): “For both vaccine and booster status models, predicted probabilities for the logistic regression model were generated using the linear predictor (i.e., the log-odds) from the logistic regression model. Continuous covariates were held constant at their means and the categorical covariates were held constant at their reference category.”**

Specify the in Figure 2 in the Y and X axis vaccine hesitancy score or booster hesitancy score or covid-19 false beliefs score. This should be considered to enable to reader understand the meaning of the variable.

**The Y-axis on the left plot is labeled “Vaccine Hesitancy”, the Y-axis on the right plot is labeled “Booster Hesitancy”, the X-axis in both plots is labeled as COVID-19 False Beliefs. Please let us know if further clarification is needed.**

Conclusion
The conclusion does not report the main findings from the study. The comparison between countries is not reported. The mains independent variables explaining vaccine uptake or acceptance are not mentioned. Kindly suggest some strategy to combat false belief in these countries especially in US. I don’t agree with the justification provided by author for not clearly aligning their conclusion with some standards.

**Thank you for this comment. We have significantly added to the Conclusion section, including the country comparison and independent variables. We have also suggested some more specific strategies to combat false beliefs (Lines 416-427).**

Reviewer #2: Everything looks good

**Thank you for reviewing the revisions to our manuscript!**
