# Supplementary material for: A cross-sectional survey of false beliefs about COVID-19 and their association with vaccine hesitancy and uptake in the United States and China
Source: PLOS Glob Public Health. 2026 Feb 25;6(2):e0005939. doi: 10.1371/journal.pgph.0005939 (PMC12935220; doi:10.1371/journal.pgph.0005939)
Supplement: Attachment — Submitted filename: Response to Reviewers R3.docx [file pgph.0005939.s004.docx]

Reviewer #1: Thank you for the revisions and congratulations.

**Thank you for taking the time to review our revised manuscript. We have addressed the remaining comments below.**

There is still formatting style issue in this manuscript in writing decimal value like .21 instead of 0.21 or < .001 instead of < 0.001. If this can be addressed by the copyediting service of the journal, it is ok for me. If not, I suggest to author to do it as

**These have all been fixed now.**

The word score is missing in the X and the Y axis of figure 2. This can be added in the footnote of this figure

**The X and Y axis of all figures are clearly labeled. We have added a legend that indicates that higher values indicate more false beliefs (figures 1 and 2) and more hesitancy (figure 2). We realized that the figures uploaded to the submission site were mislabeled (ex: figure 1a should have been figure 2a and vice versa). Formatted and correctly labeled figures have been uploaded to the submission site.**

Figure Resubmissions:

While revising your submission, we strongly recommend that you use PLOS’s NAAS tool (https://ngplosjournals.pagemajik.ai/artanalysis) to test your figure files. NAAS can convert your figure files to the TIFF file type and meet basic requirements (such as print size, resolution), or provide you with a report on issues that do not meet our requirements and that NAAS cannot fix.

After uploading your figures to PLOS’s NAAS tool - https://ngplosjournals.pagemajik.ai/artanalysis, NAAS will process the files provided and display the results in the "Uploaded Files" section of the page as the processing is complete. If the uploaded figures meet our requirements (or NAAS is able to fix the files to meet our requirements), the figure will be marked as "fixed" above. If NAAS is unable to fix the files, a red "failed" label will appear above. When NAAS has confirmed that the figure files meet our requirements, please download the file via the download option, and include these NAAS processed figure files when submitting your revised manuscript.

**We have loaded our figures into the NAAS tool and have uploaded the figures the tool fixed to meet the technical requirements.**
